# Development and validation of a novel CD4^+^ T cell‐related gene signature to detect severe COVID‐19

**DOI:** 10.1002/ctm2.1294

**Published:** 2023-06-05

**Authors:** Da‐Wei Zhang, Fang Li, Yuan‐Yuan Wei, Lei Hu, Su‐Hong Chen, Ming‐Ming Yang, Wen‐Ting Zhang, Guang‐He Fei

**Affiliations:** ^1^ Department of Respiratory and Critical Care Medicine First Affiliated Hospital of Anhui Medical University Hefei Anhui Province People's Republic of China; ^2^ Key Laboratory of Respiratory Diseases Research and Medical Transformation of Anhui Province Hefei Anhui Province People's Republic of China; ^3^ Department of Integrated Traditional Chinese and Western Medicine Anhui Medical University Hefei Anhui Province People's Republic of China

## Dear Editor,

It's well known that the coronavirus disease 2019 (COVID‐19) has posed great harm to world public health security these years.^1^ Though most patients were mild, some developed severe symptoms, especially for the elder.^2^ Therefore, a prediction model for severe COVID‐19 can identify potential severe patients and provide targeted treatment timely. The COVID‐19 severity depended significantly on the host's immune responses.^3^ CD4^+^ T cell exhaustion and decline in function are the critical immune mechanisms in the deterioration of COVID‐19.^4^, ^5^ In our study, we build a CD4^+^ T cell‐related gene signature to provide greater insight into the immune mechanisms behind COVID‐19, and it is also beneficial for the establishment of a prediction model for severe COVID‐19 and clinical therapy.

**FIGURE 1:**
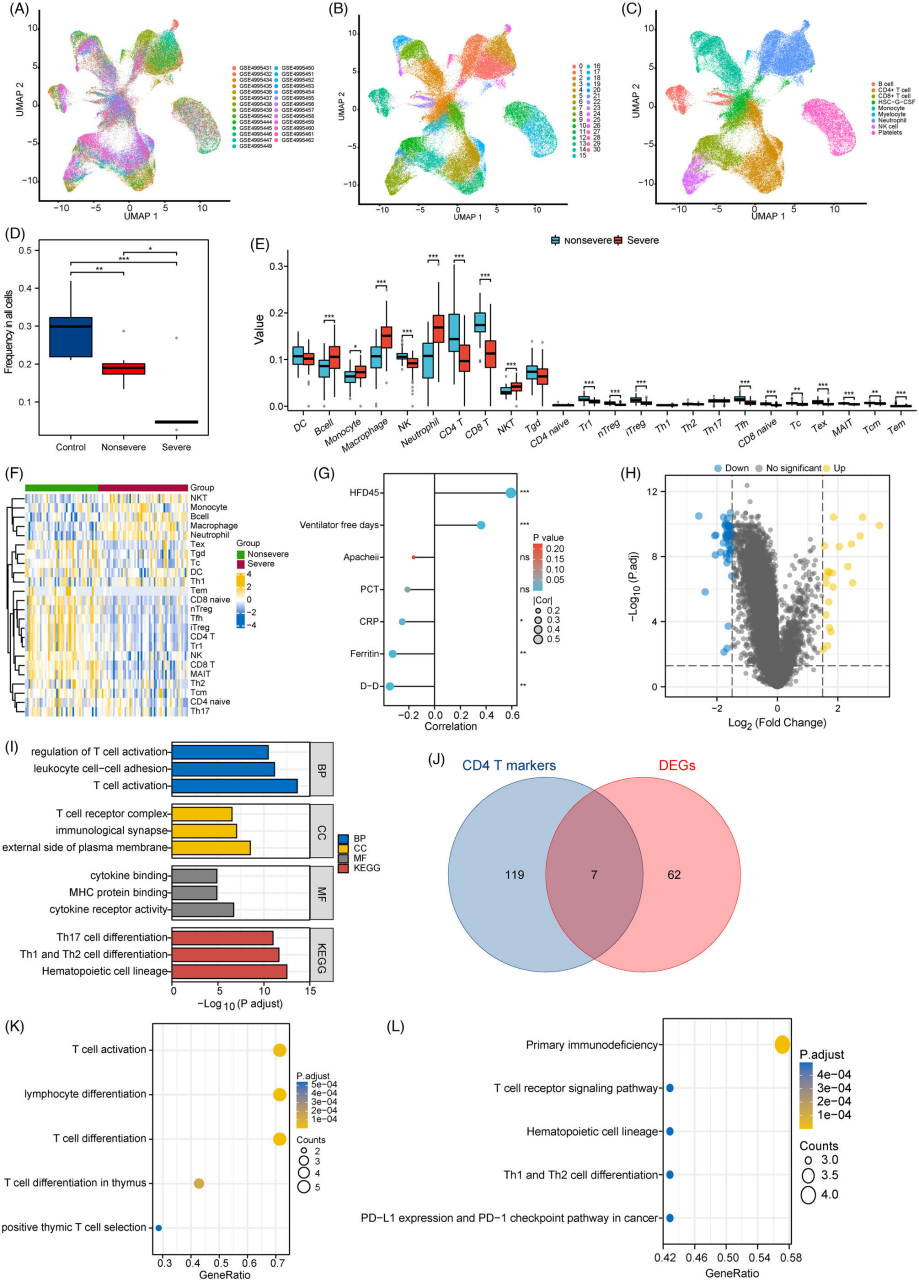
The immune landscape of coronavirus disease 2019 (COVID‐19) and identification of key differentially expressed CD4^+^ T cell markers between non‐severe COVID‐19 and severe COVID‐19 groups. (A) UMAP plot of 27 samples with and without Severe acute respiratory syndrome coronavirus 2 (SARS‐Cov‐2) in GES163668. (B) UMAP plot coloured by various cell clusters in GES163668. (C) Proportions of immune cell clusters identified by marker genes in GES163668. (D) CD4^+^ T cell frequency in COVID‐19 patients with different clinical outcomes in GES163668. (E) Comparison boxplot of 24‐immune‐cell infiltration between non‐severe COVID‐19 and severe COVID‐19 group in GES157103. (F) The heatmap of 24‐immune‐cell infiltration in non‐severe COVID‐19 and severe COVID‐19 group in GES157103. (G) Correlation of immune infiltration with clinical data. (H) Volcano plot of differentially expressed genes (DEGs) between the non‐severe group and severe group of COVID‐19 patients. (I) Gene ontology (GO) and Kyoto Encyclopedia of Genes and Genomes (KEGG) enrichment analyses of DEGs. (J) Venn diagram between DEGs and CD4^+^ T cell marker genes. (K) GO‐biological process (BP) enrichment analysis of the differentially expressed CD4^+^ T cell markers. (L) KEGG pathway enrichment analysis of the differentially expressed CD4^+^ T cell markers. ns, not significant. *p* > .05; *p < .05; **p < .01; ***p < .001.

A flowchart of this study is shown in Figure S1. Through filtering single‐cell RNA‐sequencing data of GSE163668, we acquired gene expression profiles of 100776 cells from 27 COVID‐19 samples (Figure 1A). Using the first 2000 variable genes we identified 31 cell clusters (Figure 1B) with identity annotations and clusters 5, 6, 9, 12, 18 and 30 were classified as CD4^+^ T cells (Figure 1C). Across the clusters, there were 126 genes differentially expressed, which were identified as CD4^+^ T cell marker genes (Table S1). The frequency of CD4^+^ T cell has an obvious reduction in COVID‐19 and decreased more in the severe group (Figure 1D). Hence, the GSE157103 dataset was downloaded for further analysis. The degree of CD4^+^ T cell infiltration is reduced in severe COVID‐19 (Figure 1E,F), and the CD4^+^ T infiltration score was correlated with clinical indicators related to disease severity, especially hospital‐free days at Day 45 (HFD45; Figure 1G). These outcomes are in agreement with earlier reports^6^ and indicate that CD4^+^ T cell is instrumental in the disease progression of critical COVID‐19.

**Critical points**

1. CD4^+^ T cells have a significant reduction in the severe coronavirus disease 2019 (COVID‐19) group.
2. The risk score containing CD3D, CD3E, LCK and EVL may be the prediction model of severe COVID‐19.
3. The risk score is an independent risk factor for severe COVID‐19.
4. Mitochondrial and immune function can be developed and explored as the main intervention measures to protect the function of severe COVID‐19.

To further identify key CD4^+^ T cell markers, we discovered 69 differentially expressed genes (DEGs) between non‐severe and severe patients in GSE157103 (Figure 1H, Table S2). They have enriched some T cell‐related pathways in the Kyoto Encyclopedia of Genes and Genomes (KEGG) pathway analysis. As can be seen in the biological process (BP) analysis, they were mainly connected to the regulation of T‐cell activation. Cellular component enrichment was primarily associated with the T cell receptor complex. The molecular function enrichment was related to cytokine binding (Figure 1I, Table S3). Seven genes were filed out through the intersection of DEGs and CD4^+^ T cell markers (Figure 1J). They were involved in the process of T‐cell activation in Gene ontology (GO)‐BP analysis (Figure 1K, Table S4). KEGG pathway analysis showed they were associated with primary immunodeficiency (Figure 1L, Table S4).

**FIGURE 2:**
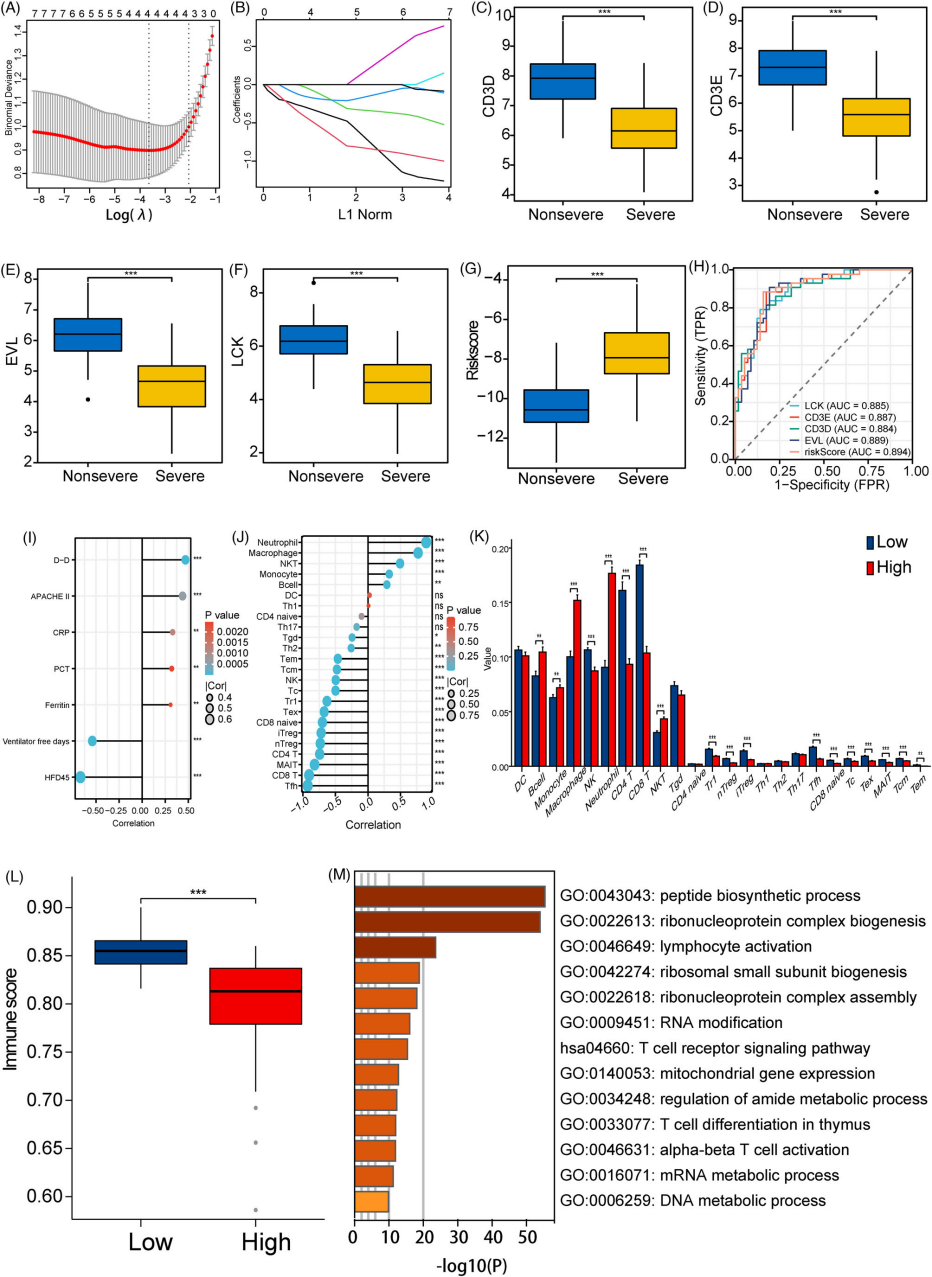
Establishment and evaluation of the gene signature in GSE157103. (A) The optimal lambda was determined when the partial likelihood of deviance reached the minimum value. (B) Least absolute shrinkage and selection operator regression (LASSO) coefficient profiles of the candidate genes for diagnostic model construction. (C) Differential expression of CD3D between non‐severe and severe patients in GSE157103. (D) Differential expression of CD3E between non‐severe and severe patients in GSE157103. (E) Differential expression of EVL between non‐severe and severe patients in GSE157103. (F) Differential expression of LCK between non‐severe and severe patients in GSE157103. (G) The distribution of risk score between the non‐severe and the severe group in GSE157103. (H) Least absolute shrinkage and selection operator regression (ROC) analysis of the four key genes and risk score for severe COVID‐19 diagnosis in GSE157103. (I) Correlation of risks core with clinical data. (J) Correlation of risk score with 24 types of immune cell infiltration. (K) Comparison boxplot of 24‐immune‐cell infiltration between low‐risk score and high‐risk score group. (L) Immune score in the low‐risk score and the high‐risk score. (M) GO‐BP and KEGG pathway enrichment analysis of risk score‐related genes. ns, not significant. *p* > .05; *p < .05; **p < .01; ***p < .001.

Given that severe COVID‐19 progresses rapidly and is difficult to rescue, it is necessary to discover an early biomarker or a model for predicting the severity of this disease. Least absolute shrinkage and selection operator regression (LASSO) analyses were conducted, and four genes were filtered out including CD3D, CD3E, LCK and EVL (Figure 2A,B). The risk score is calculated using this formula: risk score = −0.428 * CD3E − 0.693 * EVL − 0.229 *LCK − 0.204 * CD3D. It illustrated that the four key genes were decreased in severe COVID‐19 (Figure 2C,F), and the severe patients had noticeably higher risk scores (Figure 2G). As can be seen from Figure 2H, this gene signature showed good accuracy. Meanwhile, the risk score was significantly correlated with clinical indicators related to disease severity including D dimer (D‐D), acute physiology and chronic health evaluation II score, HFD45, ventilator‐free days, C‐reactive protein (CRP), procalcitonin (PCT) and ferritin (Figure 2I). The detailed baseline information is summarized in Table S5. As expected, CD4^+^ T cell infiltration was fairly correlated with the risk score (Figure 2J). We then compared the clinical data between the high‐ and low‐risk groups. More severe cases and higher ferritin, CRP, D‐D and PCT existed in the high‐risk group (Table S6). The high‐risk group also had a significant drop in CD4^+^ T infiltration score (Figure 2K). The B cells, macrophage and neutrophil infiltration scores were increased (Figure 2K), which contribute to cytokine storm in COVID‐19 patients. It indicated that the patients in the high‐risk group had a more significant imbalance of immune status (lymphocytopenia and inflammatory storm). Severe acute respiratory syndrome coronavirus 2 (SARS‐CoV‐2) is prone to errors during replication. The structural changes caused by the mutation occurred at the immune recognition site like spike protein can result in immune escape.^7^ A recent study has raised the possibility that the SARS‐CoV‐2 in immunocompromised patients may mutate to become less sensitive to neutralising antibodies when prolonged viral replication occurs.^8^ In our research, high‐risk patients had lower immune scores (Figure 2L), suggesting they might be immunocompromised and it might make the virus more susceptible to mutate.

To clarify the mechanism, we further performed an enrichment analysis of the risk score‐related genes (Table S7) and found most of them were enriched in mitochondrial BPs and metabolic pathways (Figure 2M, Table S8). This could be the underlying reason why people with metabolic dysfunction may react more severely to COVID‐19. Interestingly, other studies observed Gaucher patients, as a metabolic disease showed protection against expansion of severe form.^9^, ^10^ Therefore, the role of metabolic function played in COVID‐19 needs more research to verify. We included clinical information and risk scores in univariate and multivariate logistic regression analysis, which showed that risk score and D‐D were both independent risk factors for critical cases (Table 1). When subjects with a risk score and D‐D levels rose, they were more likely to develop severe disease.

**TABLE 1:** Univariable and multivariable logistic regression analysis of the CD4^+^ T cell‐related gene signature in GSE157103 cohort.

	**Univariable analysis**			**Multivariable analysis**		
**Variables**	**OR**	**95%CI**	***p*‐value**	**OR**	**95%CI**	***p*‐value**
**Age**						
<60	Reference			Reference		
≥60	2.778	(1.225, 6.299)	.014	2.183	(.538, 8.848)	.274
**Gender**						
Female	Reference					
Male	1.583	(.700, 3.580)	.270			
**Charlson Score**	1.134	(.958, 1.342)	.144			
**Ferritin, ng/ml**						
≤200	Reference					
>200	2.065	(.779, 5.474)	.145			
**CRP, mg/L**						
≤3	Reference					
>3	1.472	(.089, 24.306)	.787			
**D‐D, mg/L**						
≤1	Reference			Reference		
>1	14.796	(5.356, 40.872)	<.001	9.613	(2.037, 45.377)	.004
**PCT, ng/mL**						
≤.5	Reference			Reference		
>.5	4.912	(1.903, 12.677)	.001	3.913	(.943, 16.244)	.060
**LAC, mmol/L**						
≤1.7	Reference					
>1.7	2.842	(.318, 25.395)	.350			
**FIB, mg/dL**						
≤400	Reference					
>400	.487	(.142, 1.670)	.252			
**Risk score**						
Low	Reference			Reference		
High	40.533	(12.541, 131.008)	<.001	23.922	(5.585, 102.465)	<.001

Abbreviations: CI, confidence interval; CRP, C‐reactive protein; D‐D, D dimer; FIB, fibrinogen; LAC, lactate; OR, odds ratio; PCT, procalcitonin.

**FIGURE 3:**
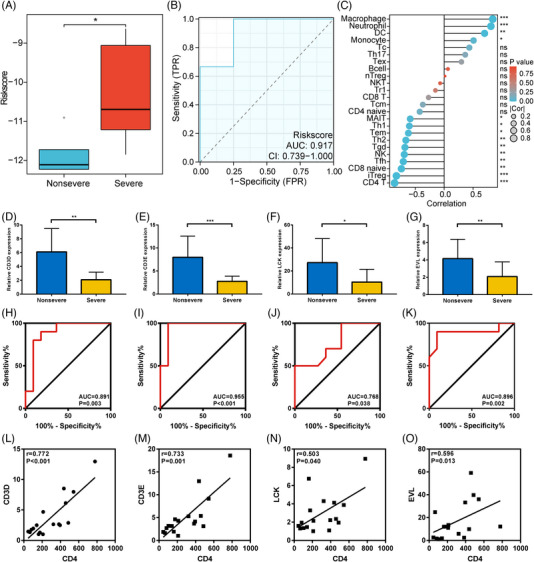
The validation of the gene signature in GSE152418 and clinical subjects. (A) The distribution of risk score between the non‐severe and the severe group in GSE152418. (B) ROC analysis of risk score for severe COVID‐19 diagnosis in GSE152418. (C) Correlation between risk score and 24 types of immune cell infiltration in GSE152418. (D) Differential expression of CD3D between non‐severe and severe patients in clinical subjects. (E) Differential expression of CD3E between non‐severe and severe patients in clinical subjects. (F) Differential expression of LCK between non‐severe and severe patients in clinical subjects. (G) Differential expression of EVL between non‐severe and severe patients in clinical subjects. (H) ROC analysis of the CD3D for severe COVID‐19 diagnosis in clinical subjects. (I) ROC analysis of the CD3E for severe COVID‐19 diagnosis in clinical subjects. (J) ROC analysis of the LCK for severe COVID‐19 diagnosis in clinical subjects. (K) ROC analysis of the EVL for severe COVID‐19 diagnosis in clinical subjects. (L) Correlation between the CD3D expression and CD4^+^ T cell counts in clinical subjects. (M) Correlation between the CD3E expression and CD4^+^ T cell counts in clinical subjects. (N) Correlation between the LCK expression and CD4^+^ T cell counts in clinical subjects. (O) Correlation between the EVL expression and CD4^+^ T cell counts in clinical subjects. ns, not significant. *p* > .05; *p < .05; **p < .01; ***p < .001.

To validate our results, we found the severe group also had high‐risk score than the non‐severe group in GSE152418 (Figure 3A). The gene signature also had a positive diagnostic efficacy for severe COVID‐19 (Figure 3B) and was related to CD4^+^ T cells (Figure 3C). Finally, we collected blood and clinical information from COVID‐19 patients. The detailed demographic characteristics are shown in Table S9. The four key genes were decreased in severe patients significantly in mRNA levels (Figure 3D–G). They also existed a good diagnostic value for severe COVID‐19 (Figure 3H–K) and a positive association with CD4^+^ T count (Figure 3L–O). However, the specific mechanism still needs investigation. The combination between clinical data and gene signature is more convincing to predict the severity of COVID‐19.

In summary, we identify a CD4^+^ T cell‐related gene signature through a combination of data from public databases and clinical COVID‐19 patient samples, shedding some light on the pathogenic mechanisms of the occurrence of severe COVID‐19. More research is warranted on the CD4^+^ T cell‐related immune processes in COVID‐19 pathology.

## CONFLICT OF INTEREST STATEMENT

The authors declare that they have no competing interests.

## FUNDING INFORMATION

Key Technology Research and Development Program of Anhui Province (No. 2022e07020076) and subject construction project of Anhui Medical University (No. 2021lcxk001).

## Supporting information

Supplementary Information

Supplementary Information

Supplementary Information

Supplementary Information

Supplementary Information

Supplementary Information

Supplementary Information

Supplementary Information

Supplementary Information

Supplementary Information
