# Supplementary material for: Development and validation of a novel CD4+ T cell‐related gene signature to detect severe COVID‐19
Source: Clin Transl Med. 2023 Jun 5;13(6):e1294. doi: 10.1002/ctm2.1294 (PMC10242253; doi:10.1002/ctm2.1294)
Supplement: Supplementary file 6 — Supplementary Information [file CTM2-13-e1294-s006.docx]

**Table S5: Demographic characteristics of COVID-19 patients in the derivation and validation cohorts in GEO datasets.**

| **Variables** | **Derivation cohorts**  **(GSE157103)** | | | **Validation cohorts**  **(GSE152418)** | | |
| --- | --- | --- | --- | --- | --- | --- |
|  | **Non-severe**  **(n=43)** | **Severe**  **(n=57)** | ***P* value** | **Non-severe**  **(n=4)** | **Severe**  **(n=12)** | ***P* value** |
| Age, years | 66.40(15.45) | 62.59(13.41) | 0.108^a^ | — | — | — |
| Mal, n (%) | 24(55.81%) | 38(66.67%) | 0.268^c^ | 0(0.00%) | 7(58.33%) | — |
| APACHE II | NA | 21.32(8.26） | — | — | — | — |
| Charlson Score | 3.70(2.75) | 3.00(4.00) | 0.172^b^ | — | — | — |
| Ventilator free days | 28.00(0.00) | 15.00(28.00) | <0.001^b^ | — | — | — |
| Sofa | NA | 7.00(5.00) | — | — | — | — |
| HFD45, days | 29.50(14.32) | 3.00(24.00) | <0.001^b^ | — | — | — |
| ICU, n (%) |  |  | <0.001^c^ |  |  | — |
| Yes | 0(0.00%) | 50(87.72%) |  | — | — |  |
| No | 43(100.00%) | 7(12.28%) |  | — | — |  |
| Mechanical ventilation, n (%) |  |  | <0.001^c^ |  |  | — |
| Yes | 0(0.00%) | 42(73.68%) |  | — | — |  |
| No | 43(100.00%) | 15(26.32%) |  | — | — |  |
| Laboratory parameters |  |  |  |  |  |  |
| Ferritin, ng/ml | 689.80(333.57) | 800.00(924.00) | 0.030^b^ | — | — | — |
| CRP, mg/l | 158.24(117.63) | 155.29(108.86) | 0.066^a^ | — | — | — |
| D-D, mg/l | 1.96(1.37) | 5.27(19.34) | <0.001^b^ | — | — | — |
| PCT, ng/ml | 1.34(1.32) | 1.10(2.47) | 0.001^b^ | — | — | — |
| LAC, mmol/l | 0.96(0.50) | 1.26(0.48) | 0.281^b^ | — | — | — |
| FIB, mg/dl | 583.30(217.85) | 538.63(222.49) | 0.675^a^ | — | — | — |

**Notes:** Data are presented as number (%) or means (standard deviation) or median (interquartile range).

**Abbreviations:** HFD45, hospital-free days at day 45; ICU, Intensive Care Unit; CRP, C-reactive protein; D-D, d dimer; PCT, procalcitonin; LAC, Lactate; FIB, Fibrinogen. ^a^t-test; ^b^Mann-Whitney U test; ^c^χ2 test.
