# Supplementary material for: Development and validation of a novel CD4+ T cell‐related gene signature to detect severe COVID‐19
Source: Clin Transl Med. 2023 Jun 5;13(6):e1294. doi: 10.1002/ctm2.1294 (PMC10242253; doi:10.1002/ctm2.1294)
Supplement: Supplementary file 7 — Supplementary Information [file CTM2-13-e1294-s003.docx]

**Table S6: Demographic characteristics of the high-risk and low-risk COVID-19 patients in the GSE157103.**

| **Variables** | **Low-risk**  **(n=47)** | **High-risk**  **(n=53)** | ***P* value** |
| --- | --- | --- | --- |
| Age, years | 61.71(14.83) | 64.15(13.34) | 0.092^a^ |
| Mal, n (%) | 26(55.32%) | 36(67.92%) | 0.195^c^ |
| APACHE II | 14.89(4.31) | 22.56(8.21) | 0.009^a^ |
| Charlson Score | 3.29(2.73) | 3.00(5.00) | 0.167^b^ |
| Ventilator free days | 28.00(14.00) | 16.50(28.00) | <0.001^b^ |
| Sofa | 5.56(2.35) | 8.00(6.00) | 0.034^b^ |
| HFD45, days | 25.00(16.67) | 1.50(22.00) | <0.001^b^ |
| Severity, n (%) |  |  | <0.001^c^ |
| Non-severe | 38(80.85%) | 5(9.43%) |  |
| severe | 9(19.15%) | 48(90.57%) |  |
| ICU, n (%) |  |  | <0.001^c^ |
| Yes | 7(14.89%) | 43(81.13%) |  |
| No | 40(85.11%) | 10(18.87%) |  |
| Mechanical ventilation, n (%) |  |  | <0.001^c^ |
| Yes | 5(10.64%) | 37(69.81%) |  |
| No | 42(89.36%) | 16((30.19%) |  |
| Laboratory parameters |  |  |  |
| Ferritin, ng/ml | 602.00(643.00) | 811.00(841.00) | 0.001^b^ |
| CRP, mg/l | 126.39(93.89) | 170.61(114.87) | 0.005^a^ |
| D-D, mg/l | 1.72(6.26) | 5.05(17.94) | <0.001^b^ |
| PCT, ng/ml | 0.98(2.63) | 1.15(2.37) | 0.022^b^ |
| LAC, mmol/l | 1.05(0.41) | 1.27(0.48) | 0.140^b^ |
| FIB, mg/dl | 537.71(209.43) | 552.24(228.25) | 0.982^a^ |

**Notes:** Data are presented as number (%) or means (standard deviation) or median (interquartile range).

**Abbreviations:** HFD45, hospital-free days at day 45; ICU, Intensive Care Unit; CRP, C-reactive protein; D-D, d dimer; PCT, procalcitonin; LAC, Lactate; FIB, Fibrinogen. ^a^t-test; ^b^Mann-Whitney U test; ^c^χ2 test.
