# Supplementary material for: Development and validation of a novel CD4+ T cell‐related gene signature to detect severe COVID‐19
Source: Clin Transl Med. 2023 Jun 5;13(6):e1294. doi: 10.1002/ctm2.1294 (PMC10242253; doi:10.1002/ctm2.1294)
Supplement: Supplementary file 10 — Supplementary Information [file CTM2-13-e1294-s007.docx]

**Table S9: Demographic characteristics of COVID-19 patients in collected samples.**

| **Variables** | **Non-severe**  **(n=11)** | **Severe**  **(n=10)** | ***P* value** |
| --- | --- | --- | --- |
| Age, years | 80.00(25.00) | 78.20(10.36) | 0.468^b^ |
| BMI, kg/m^2^ | 21.35(3.79) | 24.24(4.32) | 0.119^a^ |
| Mal, n (%) | 7(63.64) | 8(80.00) | 0.635^c^ |
| ICU, n (%) |  |  | <0.001^c^ |
| Yes | 0(0.00) | 10(100.00) |  |
| No | 11(100.00) | 0(0.00) |  |
| Mechanical ventilation, n (%) |  |  | <0.001^c^ |
| Yes | 0(0.00) | 10(100.00) |  |
| No | 11(100.00) | 0(0.00) |  |
| Laboratory parameters |  |  |  |
| WBC | 6.54(2.01) | 9.81(4.07) | 0.029^a^ |
| LYM | 1.21 (0.67) | 0.51(0.92) | 0.061^b^ |
| LYM% | 19.56(10.22) | 6.08(9.04) | 0.008^b^ |
| NEU | 4.82(1.94) | 8.60(3.95) | 0.011^a^ |
| NEU% | 72.62(10.92) | 91.76(9.89) | 0.006^b^ |
| NLR | 5.12(3.24) | 19.07(15.04) | 0.017^a^ |
| CD4 count | 421.02(162.47) | 179.70(123.85) | 0.001^a^ |
| CRP, mg/l | 43.28(42.41) | 98.30(49.31) | 0.013^a^ |
| D-D, mg/l | 1.20(1.92) | 2.13(3.02) | 0.114^b^ |
| FIB, g/l | 4.91(1.63) | 5.49(2.62) | 0.314^b^ |

**Notes:** Data are presented as number (%) or means (standard deviation) or median (interquartile range).

**Abbreviations:** ICU, Intensive Care Unit; WBC, white blood cell count; LYM, lymphocyte; LYM%, lymphocyte percentage; NEU: neutrophil count; NEU%: neutrophil percentage; NLR, Neutrophil-to-Lymphocyte Ratio; CRP, C-reactive protein; D-D, d dimer; FIB, Fibrinogen. ^a^t-test; ^b^Mann-Whitney U test; ^c^χ2 test.
